# Metastatic colon cancer of the small intestine diagnosed using genetic analysis: a case report

**DOI:** 10.1186/s13000-020-01019-6

**Published:** 2020-08-31

**Authors:** Mikiko Matsuo, Yuichiro Hatano, Yuko Imaizumi, Takahiro Kuroda, Toshinori Arai, Hiroyuki Tomita, Nobuhisa Matsuhashi, Kazuhiro Yoshida, Akira Hara

**Affiliations:** 1 Department of Tumor Pathology, Gifu University Graduate School of Medicine, 1-1 Yanagido, Gifu, 501-1194 Japan; 2 Department of Surgical Oncology, Gifu University Graduate School of Medicine, Gifu, 501-1194 Japan

**Keywords:** Small intestine, Metastatic adenocarcinoma, Colon cancer, Intestinal phenotype, *TP53*, *KRAS*, Case report

## Abstract

**Background:**

Intestinal-type adenocarcinoma is widely detected in the gastrointestinal tract, head and neck, lower respiratory and urinary systems. Determining the nature (monoclonal or multicentric) of the intestinal adenocarcinoma is sometimes a diagnostic challenge owing to its occurrence at various locations of the body, especially in the lower gastrointestinal tract. Herein, we successfully diagnosed metastatic colon cancer in the small intestine using tumor protein 53 gene (*TP53*) mutation analysis.

**Case presentation:**

An 83-year-old woman presented with severe abdominal pain and nausea at the emergency department of the hospital. Her history included surgery and adjuvant chemotherapy for colon and breast cancers. Abdominal computed tomography revealed small intestinal dilation, which was associated with the mural nodule detected on fluorodeoxyglucose positron emission tomography. Laparoscopy-assisted small bowel resection was performed based on the diagnosis of small bowel obstruction, probably due to recurrence of the colon or breast cancer. Macroscopically, an ulcerated tumor was present in the resected small intestine. Histologically, the cancer cells showed infiltrative growth of colonic dysplastic glands, whose non-specific finding made it difficult to determine the relationship with past colon cancers. Retrospective pathological examination confirmed that the previous breast and colon carcinomas were primary cancers. Immunohistochemical analysis revealed that the small intestinal and colon cancer cells showed diffuse positive tumor protein 53 (p53) expression. However, the breast cancer cells showed only weakly positive p53 expression. In addition, *TP53* mutational analysis detected an identical missense mutation (p.T211I) between the two intestinal cancers. Moreover, further molecular genetic work-up revealed that both small intestinal and colon adenocarcinomas harbored an identical missense mutation (p.G12D) of *KRAS* gene. In conclusion, the small intestinal cancer in this case was identified as a metastatic adenocarcinoma arising from a past colon cancer.

**Conclusions:**

Genetic analyses help in clarifying the identity of the cells in multiple cancer cases. In morphologically indeterminate cases, molecular analysis of common cancer-related genes can be useful for a precise and reproducible diagnosis.

## Background

Histology of cancer cells shows cell differentiation and the neoplastic process. Accordingly, unique tumor morphology, which shows its histopathological type and expected tumorigenesis, is a diagnostic tool to identify its primary site. For example, colorectal cancer is generally classified as adenocarcinoma NOS (not otherwise specified) because it resembles normal intestinal crypts or conventional colonic adenoma [1]. However, the colonic or enteric subtype is also found in other tumor classifications, including head and neck, lung, and urinary tract cancers [2–4]. Consequently, the intestinal phenotype in cancer does not always originate from the lower gastrointestinal tract. In addition, distinguishing whether multiple colonic adenocarcinomas developed from single or multicentric tumor-initiating cells can be a diagnostic challenge. Although cancer predispositions such as genetic and inflammatory factors accelerate multicentric tumor formation [5–8], these clues are sometimes hidden in the practical diagnostic setting.

Herein, we report a case of adenocarcinoma in the small intestine diagnosed with immunohistochemical and genetic analyses, which also clarified the relationship of this adenocarcinoma with past breast and colon cancers.

## Case presentation

### Clinical history

**Fig. 1:**
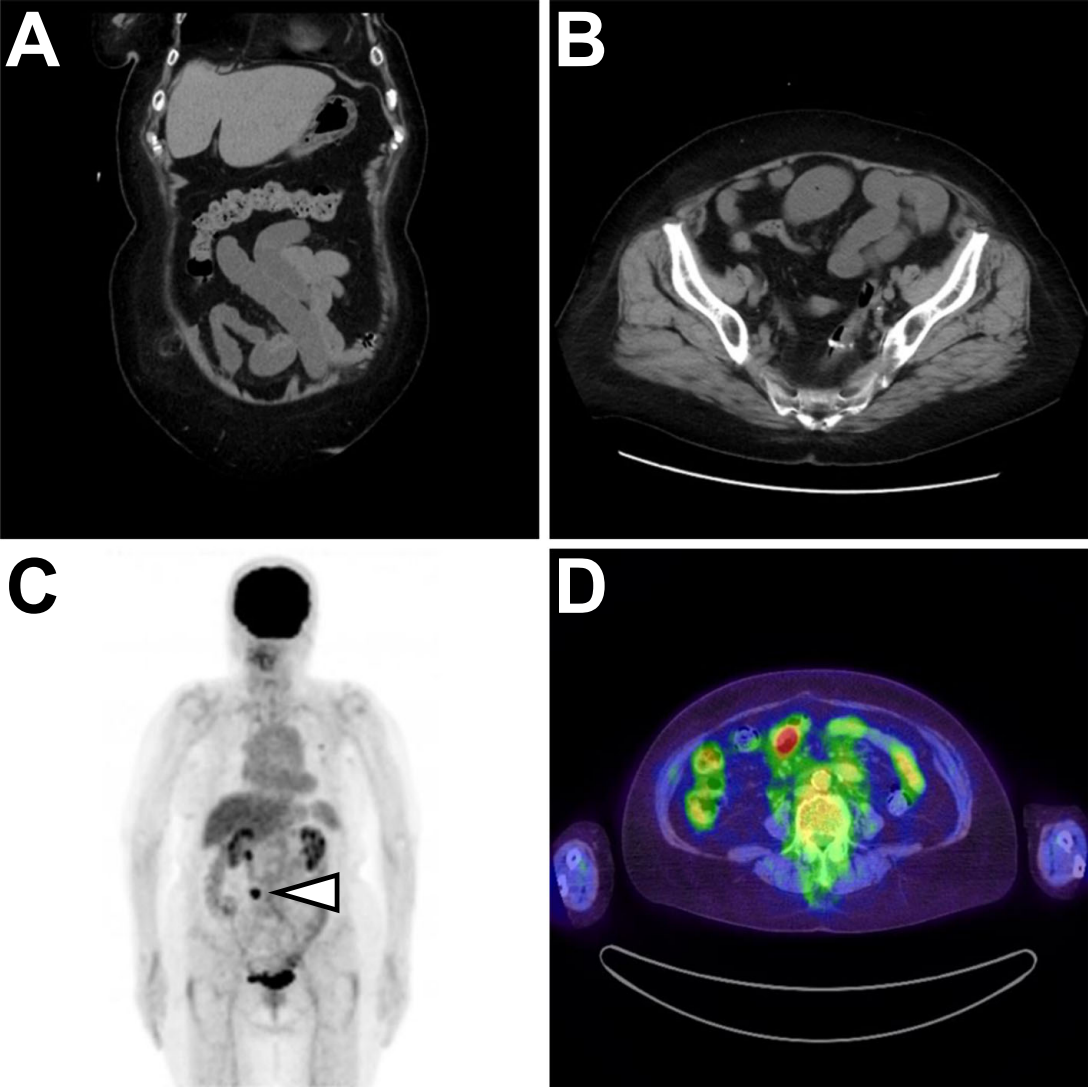
Radiological findings of the small intestinal tumor. **a** Coronal and **b** axial sections of the computed tomography scan. **c** Fluorodeoxyglucose positron emission tomographic scan showing an abdominal nodule (arrowhead). **d** The nodule is located on the small intestinal wall. The maximum standardized uptake value of the nodule was 12.81

An 83-year-old woman presented with severe abdominal pain and nausea at the emergency department of the hospital. She had undergone sigmoidectomy, followed by total mastectomy of the left breast 2 years ago. Pathological examination revealed that each lesion was a primary cancer; the colon cancer was a moderately differentiated adenocarcinoma (pT4aN0M0), whereas the breast cancer was an invasive ductal carcinoma with apocrine differentiation (pT2N1M0). After mastectomy, she received follow-up care, which included six cycles of adjuvant chemotherapy consisting of cyclophosphamide, methotrexate, and fluorouracil. In the emergency room, she was treated with scopolamine butylbromide because abdominal computed tomography (CT) showed mild dilation of the small intestine (Fig. 1a, b); she went home showing no symptoms. The next day, she returned to the hospital with relapse of the abdominal symptoms. The in-house radiological department noticed that the previous CT images showed an obstructed ileus arising from the nodule detected on a ^18^F-fluorodeoxyglucose positron emission tomography scan 3 months ago (Fig. 1c, d). No postoperative adhesion or constriction seemed to be related to the bowel obstruction. Radiological findings and history led to the diagnosis of small bowel obstruction due to the mural nodule, which probably recurred from the colon or breast cancer. Subsequently, she was admitted to the digestive surgery department and received laparoscopy-assisted small bowel resection.

### Pathological findings

**Fig. 2:**
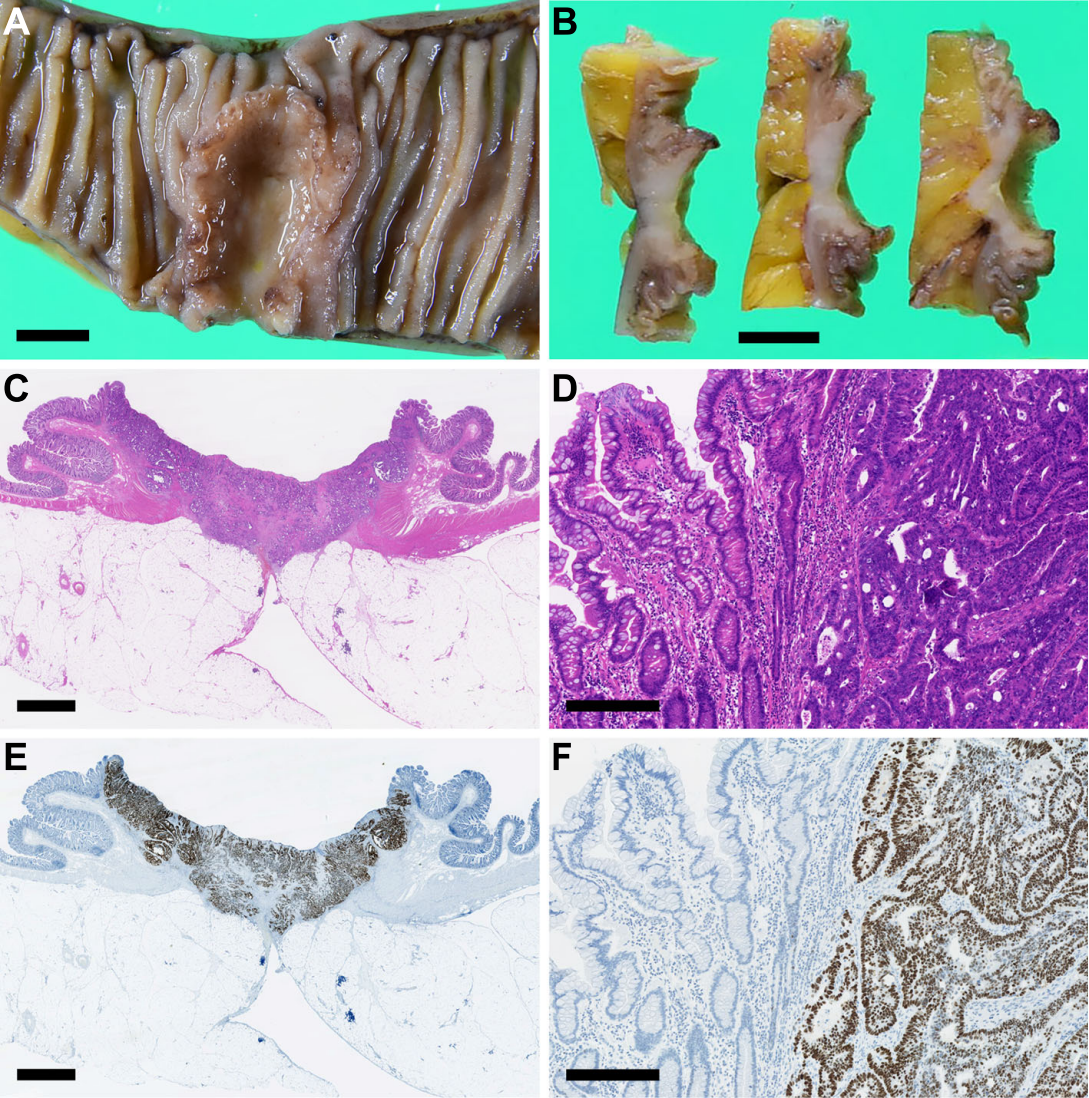
The small intestinal tumor. **a** Surface and **b** slices of the tumor. **c** A representative whole-slide and **d** magnified hematoxylin and eosin staining images of the tumor. **e** A representative whole and **f** detailed images of p53 immunostaining. Black bars: 1 cm (**a**, **b**), 2.5 mm (**c**, **e**), 250 μm (**d**, **f**)

**Fig. 3:**
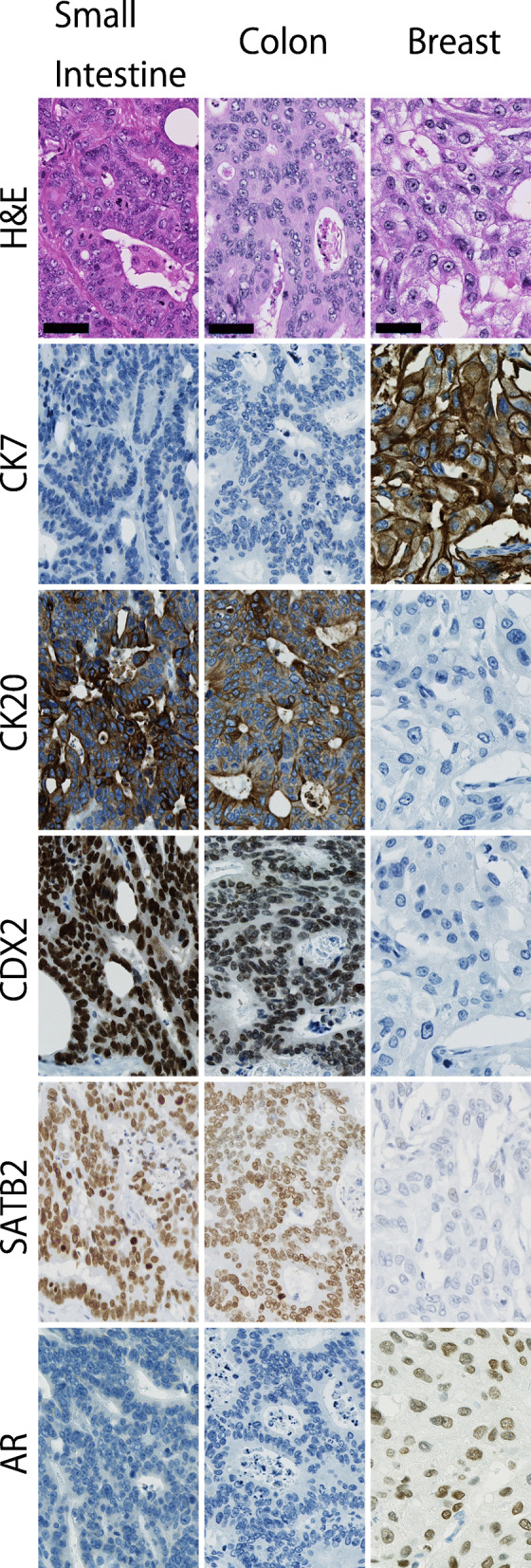
Expression of cytokeratins 7/20 and transcriptional factors in the small intestinal, colon, and breast cancer. Black bars: 50 μm

Macroscopically, the resected small intestine was found to contain an ulcerated tumor (Fig. 2a), which was located 170 cm from the ligament of Treitz. Slices of the tumor suggested that the estimated tumor depth was up to the serosal surface of the intestinal wall (Fig. 2b). Histologically, infiltrative growth of colonic dysplastic glands was observed (Fig. 2c, d). Immunohistochemically, tumor cells were diffusely positive for tumor protein 53 (p53) (Fig. 2e, f), caudal-type homeobox 2 (CDX2) and special AT-rich sequence-binding protein 2 (SATB2) (Fig. 3), positive for cytokeratin 20 (CK20) (Fig. 3) and negative for cytokeratin 7 (CK7), androgen receptor (AR) (Fig. 3), gross cystic disease fluid protein 15 (GCDFP-15), estrogen receptor (ER), progesterone receptor (PgR), and human epidermal growth factor receptor 2 (HER2). Collectively, these findings were indicative of intestinal rather than mammary gland differentiation of the tumor cells. Thus, this lesion seemed to be compatible with metastatic colon cancer, albeit its gross and histological appearance mimicking primary small intestinal cancer.

To investigate the origin of the cancer cells, we reviewed the preparation of the past surgical specimens. The immunohistochemical findings of the small intestinal, colon, and breast cancer are summarized in Table 1.

**Table 1:** Summary of immunohistochemistry in the present case

Antibody	Clone	Manufacturer	Small intestinal cancer	Colon cancer	Breast cancer
p53	DO-7	DAKO	Diffuse positive	Diffuse positive	Partial positive
CK7	OV-TL 12/30	DAKO	Negative	Negative	Positive
CK20	Ks 20.8	DAKO	Positive	Positive	Negative
CDX2	DAK-CDX2	DAKO	Diffuse positive	Diffuse positive	Negative
SATB2	EPNCIR130A	abcam	Diffuse positive	Diffuse positive	Negative
ER	SP1	Roche	Negative	Negative	Negative
PgR	1E2	Roche	Negative	Negative	Negative
HER2	4B5	Roche	Negative	Negative	Negative
AR	AR441	DAKO	Negative	Negative	Diffuse positive
GCDFP-15	23A3	Leica	Negative	Negative	Diffuse positive
Ki-67	MIB-1	DAKO	80%	40%	10%

**Fig. 4:**
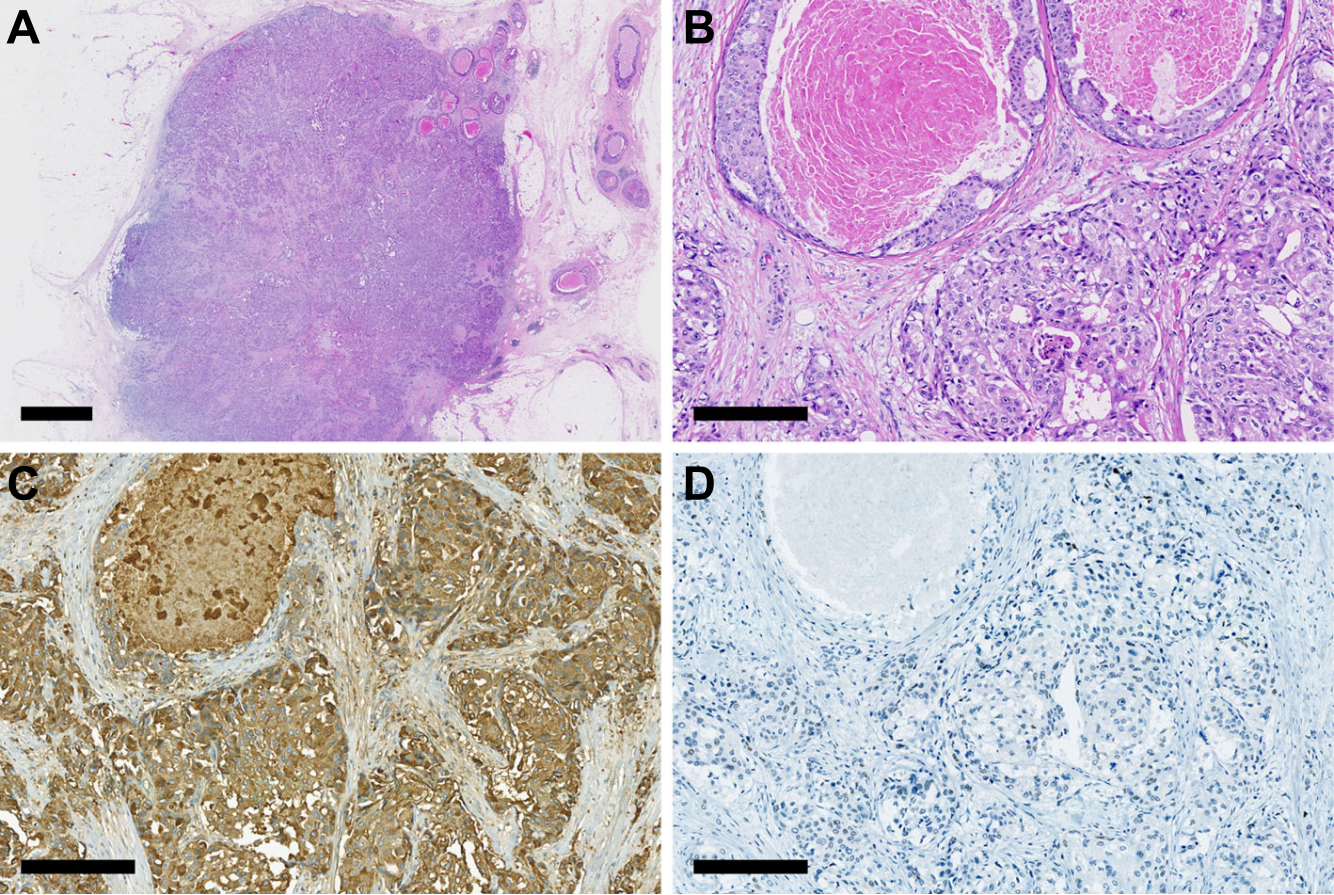
Image of a previous breast cancer. **a** Representative whole-slide and **b** magnified hematoxylin and eosin staining images of the tumor. **c** Representative images of GCDFP-15 and **d** p53 immunostaining. Black bars: 2.5 mm (**a**), 250 μm (**b**-**d**)

**Fig. 5:**
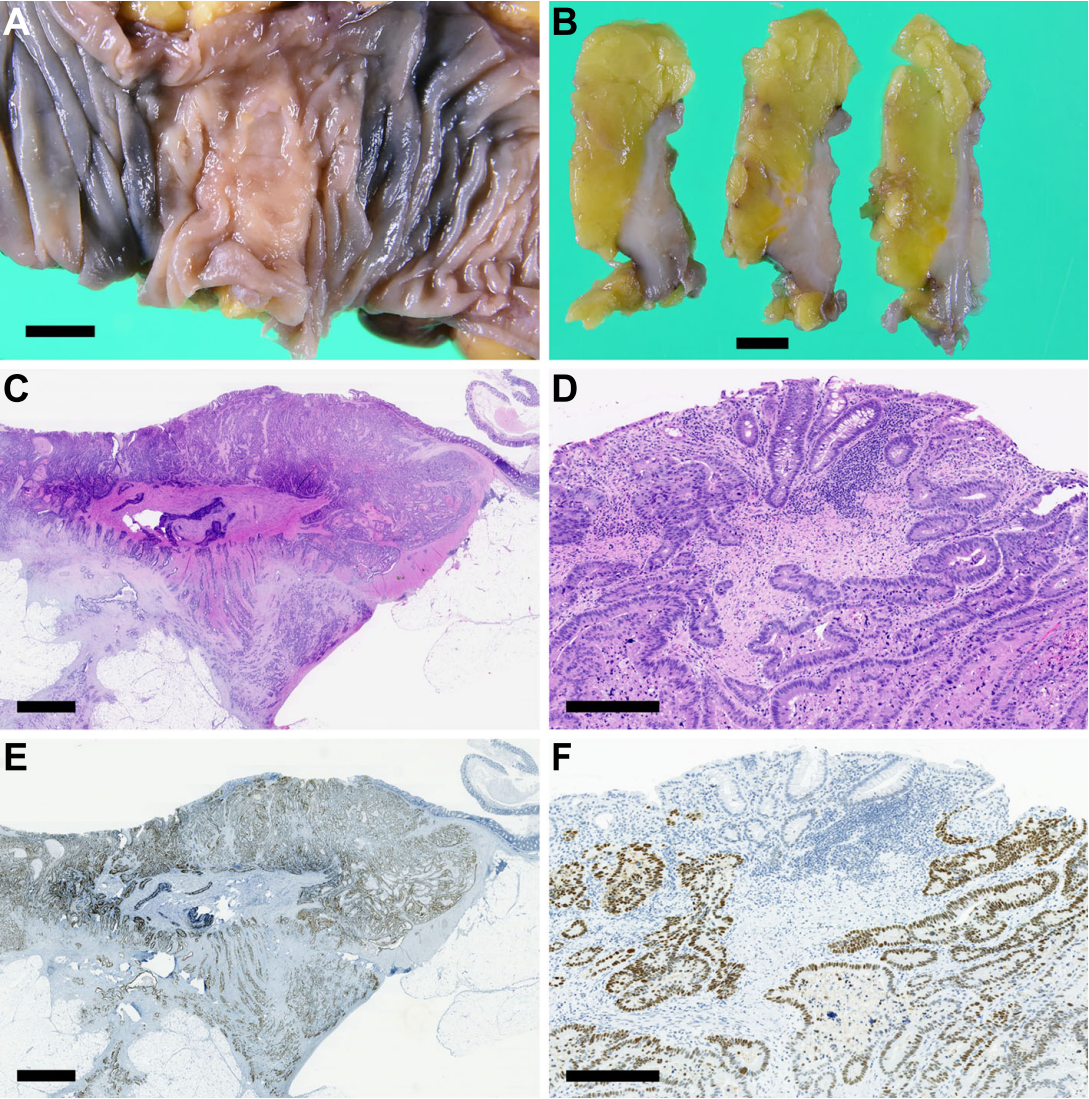
Image of a previous colon cancer. **a** Surface and **b** slices of the tumor. **c** Representative whole-slide and **d** magnified hematoxylin and eosin staining images of the tumor. **e** Representative whole-slide and **f** magnified images of p53 immunostaining. Black bars: 1 cm (**a**, **b**), 2.5 mm (**c**, **e**), 250 μm (**d**, **f**)

The breast cancer specimen (Fig. 4a) consisted of glandular and nested cells with high-grade nuclear atypia and eosinophilic granule-containing abundant cytoplasm (Fig. 4b). Immunohistochemically, the breast cancer cells were diffusely positive for AR (Fig. 3) and GCDFP-15 (Fig. 4c), positive for CK7 (Fig. 3), weakly and partially positive for p53 (Fig. 4d), and negative for CK20, CDX2, SATB2 (Fig. 3), ER, PgR, and HER2. On the other hand, the colon cancer was an ulcerated tumor (Fig. 5a) with diffuse bowel wall thickening (Fig. 5b). The histology of the tumor was compatible with typical colonic adenocarcinoma (Fig. 5c, d). Furthermore, the immunohistochemical findings of colon cancer were quite similar with those of small intestinal cancer, except for Ki-67 expression (Fig. 3, Table 1).

**Fig. 6:**
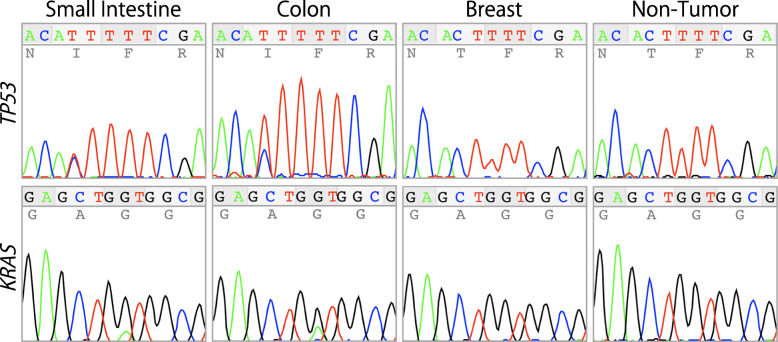
Genetic analyses of the present case. A DNA sequence analysis of *TP53* exon 6 (upper panels) and *KRAS* exon 2 (lower panels) from the small intestinal, colon, and breast carcinoma lesions and normal intestinal tissue (non-tumor). In the sequences of colon and small intestinal carcinomas, missense mutations were detected at c.632C > T in the *TP53* gene and at c.35G > A in the *KRAS* gene

As a similar abnormal p53 immunophenotype was found between the small intestinal cancer (Fig. 2e, f) and the colon cancer (Fig. 5e, f), we analyzed the *TP53* mutation status of these two tumors by direct sequencing, as described previously with minor modifications [9–11]. Consistent with the immunohistochemical findings, both cancers harbored an identical missense mutation, which was located on the codon 211 of the *TP53* gene (Fig. 6). Therefore, we concluded that the small intestinal cancer in the present case was a metastatic adenocarcinoma arising from a past colon cancer.

Additional molecular tests were then performed in order to check the status of colon cancer biomarkers in the relapsed lesion. A PCR-based *RAS*/*BRAF* genetic test revealed *KRAS* G12D mutation in the small intestinal tumor, whereas the microsatellite instability test rendered a negative result. Owing to these results, we decided to investigate the genetic status of the *KRAS* gene in the three cancers by direct sequencing [12]. Consistent with the previous molecular findings, both the small intestinal and colon cancer specimens harbored the G12D mutation, whereas the breast cancer specimen only harbored wild type alleles (Fig. 6).

The patient is alive and under watchful waiting, 18 months after the last surgery.

## Discussion

Multiple cancers that are histologically similar can be a diagnostic problem, regardless of the detection time. Similarly, multiple advanced cancers in the same organ and/or system can make pathological examination difficult. Such morphological and anatomical similarities sometimes conceal the origin of the tumor cells. Fundamentally, confirmation of the identity of multiple cancers, including precise tumor stage and pathogenesis, is important to not only satisfy scientific interest but also provide practical information for future therapeutic strategies, including choice of optimal molecular agents. Macroscopically, the small intestinal tumor in the present case was an ulcer-like lesion; histologically, it appeared as a conventional colonic adenocarcinoma. Although these findings were consistent with those of primary small intestinal cancer, its p53 immunophenotype and *TP53* mutational type were identical to those of the past colon cancer. Thus, we confirmed that these two tumors are metachronous intestinal cancer lesions, but they originated from identical neoplastic clones.

P53, which is encoded by the *TP53* gene, is associated with one of the ten canonical oncogenic signaling pathways [13]. The p53 pathway plays an important role in cell survival, proliferation, senescence, and apoptosis. Recently, a comprehensive genome analysis revealed that the *TP53* gene mutation is the most frequent genetic alteration across various cancer types [14]. It is found in approximately 60% of colon cancers [13, 15]. Notably, the known genetic alteration in the *TP53* gene consists of several hotspots and a myriad of minor sequence variants [16, 17]. This suggests that the occurrence of *TP53* somatic mutations in multiple cancers is a potent genetic signature of the identical cancer clone.

To understand the relationship between the aberrant p53 expression and *TP53* mutation, a high-grade serous carcinoma of the female genital tract was studied as a representative cancer model [18]. This kind of malignancy almost always shows an aberrant p53 expression, consistent with that of the mutated type of the *TP53* gene. Diffusely positive p53 expression principally corresponds to a missense mutation of the DNA-binding domains (exon 4–8) in the *TP53* gene, whereas diffusely negative p53 expression largely matches the frameshift, nonsense, and splicing site mutations of the *TP53* gene [19]. In addition, the third minor immunophenotype, cytoplastic p53 expression, probably occurs due to a mutation in the nuclear localization site. Collectively, the aberrant p53 immunophenotype patterns precisely predict *TP53* mutation and its mutational type.

p53 immunohistochemistry is an effective tool to distinguish *TP53*-mutated tumors; hence, an identical p53 pattern in the two tumors of the individual indicates probable monoclonal tumor origin [20]. However, the aberrant pattern of p53 expression is the only surrogate marker for the *TP53* mutational test, and the variety of the aberrant pattern is limited. Therefore, confirmation of *TP53* sequence analysis is desirable, and we successfully demonstrated the genetic link between the two intestinal cancers through the identical *TP53* mutational pattern.

At present, therapeutic planning for colon cancer requires the status of several established predictive biomarkers, including, *RAS* genes, *BRAF*, microsatellite instability [1]. Cancers with mutation of *RAS* and *BRAF* genes were found to be resistant to anti-EGFR (epidermal growth factor receptor) therapy [21, 22]. In contrast, cancers with microsatellite instability respond to anti-PDL1 therapy [23, 24]. Therefore, confirmation of these statuses is essential to select optimal molecular therapeutic agents, especially in advanced and/or relapsed colon cancer. In the present case, small intestinal and colon cancer specimens harbored identical *KRAS* mutations, suggesting that finding aberrant predictive biomarkers is also a potent diagnostic strategy to determine whether the multiple cancers derived from a single clone.

Apart from the genetic findings, immunohistochemical analysis is also useful to detect the origin of the cancer. Classically, a combination of the cytokeratins 7/20 expression has been used for assessing cancers with uncertain primary site. Most colon cancers are CK20 positive and CK7 negative, whereas most breast cancers are CK20 negative and CK7 positive [25]. Interestingly, small intestinal cancers frequently express CK7 and lack CK20 [26], despite the intestinal-type morphology. Although this aberrant immunophenotype may help in predicting the origin of the intestinal-type adenocarcinoma, presence of atypical CK7 positive and/or CK20 negative patterns are also observed in approximately a quarter of mismatch repair deficient colon cancers [27].

Alternatively, the expression of lineage-specific transcriptional factors and biomarkers help to presume cellular differentiation in cancer. A homeobox protein, CDX2, also known as a representative regulator of intestinal differentiation [28, 29] is a sensitive and specific marker of colorectal adenocarcinoma [30]. However, high grade, mucinous and/or mismatch repair deficient colonic adenocarcinomas are associated with negative CDX2 expression [27, 30], which is a prognostic factor of colon cancer without metastatic lesions [31]. Similarly, a lack of CDX2 expression is sometimes observed in small intestinal adenocarcinomas [32]. Therefore, in cases with a low CDX2 expression in intestinal-type adenocarcinomas, we should be careful about the uncommon situation mentioned above.

Recently, SATB2 is emerging as a next-generation marker for gastrointestinal tract differentiation. In addition, this nuclear matrix protein is also a marker for osteoblastic differentiation [33] because of its ability to induce skeletogenesis [34]. The expression of SATB2 is more specific to adenocarcinoma of the lower gastrointestinal tract origin compared to the expression of CDX2 [35]. However, reduced SATB2 expression (similar to that of CDX2) has been reported in mismatch repair deficient colon adenocarcinomas [36] and small intestinal cancers [37]. Taking these evidences into consideration, it is difficult to completely distinguish between colon and small intestinal cancers solely by the expression statuses of CDX2 and/or SATB2.

In contrast, AR is an emerging biomarker for prostate cancers [38], salivary duct carcinomas [39], and breast cancers [40]. Expression of this male hormone receptor in these cancers arise from AR gene dysregulation, including mutation, amplification, and alternative splicing. Consequently, an abnormal AR expression leads to cancer cell proliferation [40] even in androgen depleted states [38]. Expression of AR is significantly associated with apocrine differentiation of salivary duct carcinomas [41] and breast cancers [42] indicating that AR is a surrogate marker for these histological types [43, 44], and possess a promising therapeutic target [45].

Colorectal cancer is a leading lethal malignancy, and the most common type of cancer occurs in the gastrointestinal tract [46]. In contrast, small intestinal cancer comprises only a small fraction of human neoplasia [47]. Furthermore, metastatic lesions in the small intestine, especially the distal location, outnumber primary small intestinal cancer [1]. Considering these facts, we cannot confirm the diagnosis of primary small intestinal cancer, until the possibility of a metastatic lesion from another anatomical site is ruled out. This clinical information may help the physician to decide whether the small intestinal lesion is truly a primary cancer. In addition, gross and microscopic findings of the tumor are clues to its origin. However, a single clinical or morphological feature does not enable us to determine the identity of multiple neoplastic lesions definitively [48], as in the present case. To understand the evolutional history of cancer in individual cases, we propose that a molecular test must be conducted during each pathological examination. We believe that the molecular signatures, which consist of the genomic alterations, could properly confirm the identity of multiple cancers.

In conclusion, mutation analysis is a potent diagnostic tool to identify whether a tumor specimen is primary or secondary, regardless of the morphological features. Currently, cancer genetic analyses using next-generation sequencing are essential to find actionable molecular targets. In addition to their use in therapeutic strategies, the cancer genomic data indicate traceable molecular signatures that identify cancer cells. In the future, genomic findings could assist in the pathological diagnosis of morphologically indeterminate cases.

## Data Availability

The datasets used and/or analyzed during the current study are available from the corresponding author upon reasonable request.

## Abbreviations

AR: Androgen receptor
CDX2: Caudal-type homeobox 2
CK7: Cytokeratin 7
CK20: Cytokeratin 20
CT: Computed tomography
EGFR: Epidermal growth factor receptor
ER: Estrogen receptor
GCDFP-15: Gross cystic disease fluid protein 15
HER2: Human epidermal growth factor receptor 2
NOS: Not otherwise specified
p53: Tumor protein 53
PgR: Progesterone receptor
SATB2: Special AT-rich sequence-binding protein 2
TP53: Tumor protein 53 gene

## References

[1] Digestive system tumors. 5th ed. Lyon: WHO Press; 2019.

[2] El-Naggar AK, Chan JKC, Grandis JR, Takata T, Slootweg PJ (2017). WHO classification of head and neck tumours.

[3] Moch H, Humphrey PA, Ulbright TM, Reuter VE (2016). WHO classification of tumours of the urinary system and male genital organs.

[4] Travis WD, Brambilla E, Burke AP, Marx A, Nicholson AG (2015). WHO classification of tumours of lung, pleura, thymus and heart.

[5] Moertel CG (1977). Multiple primary malignant neoplasms: historical perspectives. Cancer.

[6] Hatano Y, Tamada M, Matsuo M, Hara A (2020). Molecular trajectory of BRCA1 and BRCA2 mutations. Front Oncol.

[7] Ma H, Brosens LAA, Offerhaus GJA, Giardiello FM, de Leng WWJ, Montgomery EA (2018). Pathology and genetics of hereditary colorectal cancer. Pathology.

[8] Dulai PS, Sandborn WJ, Gupta S (2016). Colorectal cancer and dysplasia in inflammatory bowel disease: a review of disease epidemiology, pathophysiology, and management. Cancer Prev Res (Phila).

[9] Singh N, Faruqi A, Kommoss F, McCluggage WG, Trevisan G, Senz J (2018). Extrauterine high-grade serous carcinomas with bilateral adnexal involvement as the only two disease sites are clonal based on tp53 sequencing results: implications for biology, classification, and staging. Mod Pathol.

[10] Hatano Y, Fukuda S, Makino H, Tomita H, Morishige KI, Hara A (2018). High-grade serous carcinoma with discordant p53 signature: report of a case with new insight regarding high-grade serous carcinogenesis. Diagn Pathol.

[11] Hatano Y, Tamada M, Asano N, Hayasaki Y, Tomita H, Morishige KI (2019). High-grade serous ovarian carcinoma with mucinous differentiation: report of a rare and unique case suggesting transition from the “SET” feature of high-grade serous carcinoma to the “STEM” feature. Diagn Pathol.

[12] Margonis GA, Kim Y, Spolverato G, Ejaz A, Gupta R, Cosgrove D (2015). Association between specific mutations in KRAS codon 12 and colorectal liver metastasis. JAMA Surg.

[13] Sanchez-Vega F, Mina M, Armenia J, Chatila WK, Luna A, La KC, et al. Oncogenic signaling pathways in the cancer genome atlas. Cell. 2018;173:321–37.e10. 10.1016/j.cell.2018.03.035 PMC6070353 29625050

[14] Zehir A, Benayed R, Shah RH, Syed A, Middha S, Kim HR (2017). Mutational landscape of metastatic cancer revealed from prospective clinical sequencing of 10,000 patients. Nat Med.

[15] Cancer Genome Atlas Network. Comprehensive molecular characterization of human colon and rectal cancer. Nature. 2012;487:330–7. 10.1038/nature11252 PMC3401966 22810696

[16] Bouaoun L, Sonkin D, Ardin M, Hollstein M, Byrnes G, Zavadil J (2016). TP53 variations in human cancers: new lessons from the IARC TP53 database and genomics data. Hum Mutat.

[17] Nakayama M, Oshima M (2019). Mutant p53 in colon cancer. J Mol Cell Biol.

[18] Hatano Y, Hatano K, Tamada M, Morishige KI, Tomita H, Yanai H (2019). A comprehensive review of ovarian serous carcinoma. Adv Anat Pathol.

[19] Köbel M, Piskorz AM, Lee S, Lui S, LePage C, Marass F (2016). Optimized p53 immunohistochemistry is an accurate predictor of TP53 mutation in ovarian carcinoma. J Pathol Clin Res.

[20] Kojima S, Sakamoto T, Nagai Y, Honda M, Ogawa F (2018). Metachronous rectal metastasis from primary transverse colon cancer: a case report. Surg Case Rep.

[21] Lievre A, Bachet JB, Le Corre D, Boige V, Landi B, Emile JF (2006). KRAS mutation status is predictive of response to cetuximab therapy in colorectal cancer. Cancer Res.

[22] Sepulveda AR, Hamilton SR, Allegra CJ, Grody W, Cushman-Vokoun AM, Funkhouser WK (2017). Molecular biomarkers for the evaluation of colorectal cancer: guideline summary from the American Society for Clinical Pathology, College of American Pathologists, Association for Molecular Pathology, and American Society of Clinical Oncology. J Oncol Pract.

[23] Dudley JC, Lin MT, Le DT, Eshleman JR (2016). Microsatellite instability as a biomarker for PD-1 blockade. Clin Cancer Res.

[24] Alvi MA, Loughrey MB, Dunne P, McQuaid S, Turkington R, Fuchs MA (2017). Molecular profiling of signet ring cell colorectal cancer provides a strong rationale for genomic targeted and immune checkpoint inhibitor therapies. Br J Cancer.

[25] Chu P, Wu E, Weiss LM (2000). Cytokeratin 7 and cytokeratin 20 expression in epithelial neoplasms: a survey of 435 cases. Mod Pathol.

[26] Chen ZE, Wang HL. Alteration of cytokeratin 7 and cytokeratin 20 expression profile is uniquely associated with tumorigenesis of primary adenocarcinoma of the small intestine. Am J Surg Pathol. 2004;28:1352–9. 10.1097/01.pas.0000135520.72965.50 15371952

[27] Lugli A, Tzankov A, Zlobec I, Terracciano LM (2008). Differential diagnostic and functional role of the multi-marker phenotype CDX2/CK20/CK7 in colorectal cancer stratified by mismatch repair status. Mod Pathol.

[28] Kumar N, Tsai YH, Chen L, Zhou A, Banerjee KK, Saxena M (2019). The lineage-specific transcription factor CDX2 navigates dynamic chromatin to control distinct stages of intestine development. Development.

[29] Hatano Y, Semi K, Hashimoto K, Lee MS, Hirata A, Tomita H (2015). Reducing DNA methylation suppresses colon carcinogenesis by inducing tumor cell differentiation. Carcinogenesis.

[30] Kaimaktchiev V, Terracciano L, Tornillo L, Spichtin H, Stoios D, Bundi M (2004). The homeobox intestinal differentiation factor CDX2 is selectively expressed in gastrointestinal adenocarcinomas. Mod Pathol.

[31] Dalerba P, Sahoo D, Paik S, Guo X, Yothers G, Song N (2016). CDX2 as a prognostic biomarker in stage II and stage III colon cancer. N Engl J Med.

[32] Zhang MQ, Lin F, Hui P, Chen ZE, Ritter JH, Wang HL. Expression of mucins, SIMA, villin, and CDX2 in small-intestinal adenocarcinoma. Am J Clin Pathol. 2007;128:808–16. 10.1309/JAF3KVGJHQCJ1QF9 17951204

[33] Conner JR, Hornick JL (2013). SATB2 is a novel marker of osteoblastic differentiation in bone and soft tissue tumours. Histopathology.

[34] Dobreva G, Chahrour M, Dautzenberg M, Chirivella L, Kanzler B, Farinas I (2006). SATB2 is a multifunctional determinant of craniofacial patterning and osteoblast differentiation. Cell.

[35] Brettfeld SM, Ramos BD, Berry RS, Martin DR, Hanson JA (2019). SATB2 versus CDX2: a battle royale for diagnostic supremacy in mucinous tumors. Arch Pathol Lab Med.

[36] Ma C, Olevian D, Miller C, Herbst C, Jayachandran P, Kozak MM (2019). SATB2 and CDX2 are prognostic biomarkers in DNA mismatch repair protein deficient colon cancer. Mod Pathol.

[37] Kim CJ, Baruch-Oren T, Lin F, Fan XS, Yang XJ, Wang HL (2016). Value of SATB2 immunostaining in the distinction between small intestinal and colorectal adenocarcinomas. J Clin Pathol.

[38] Fujita K, Nonomura N (2019). Role of androgen receptor in prostate cancer: a review. World J Mens Health.

[39] Udager AM, Chiosea SI (2017). Salivary duct carcinoma: an update on morphologic mimics and diagnostic use of androgen receptor immunohistochemistry. Head Neck Pathol.

[40] Giovannelli P, Di Donato M, Galasso G, Di Zazzo E, Bilancio A, Migliaccio A (2018). The androgen receptor in breast cancer. Front Endocrinol (Lausanne).

[41] Dalin MG, Desrichard A, Katabi N, Makarov V, Walsh LA, Lee KW (2016). Comprehensive molecular characterization of salivary duct carcinoma reveals actionable targets and similarity to apocrine breast cancer. Clin Cancer Res.

[42] Robinson JL, Macarthur S, Ross-Innes CS, Tilley WD, Neal DE, Mills IG (2011). Androgen receptor driven transcription in molecular apocrine breast cancer is mediated by FoxA1. EMBO J.

[43] Williams EM, Higgins JP, Sangoi AR, McKenney JK, Troxell ML (2015). Androgen receptor immunohistochemistry in genitourinary neoplasms. Int Urol Nephrol.

[44] Vranic S, Tawfik O, Palazzo J, Bilalovic N, Eyzaguirre E, Lee LM (2010). EGFR and HER-2/neu expression in invasive apocrine carcinoma of the breast. Mod Pathol.

[45] Mitani Y, Rao PH, Maity SN, Lee YC, Ferrarotto R, Post JC (2014). Alterations associated with androgen receptor gene activation in salivary duct carcinoma of both sexes: potential therapeutic ramifications. Clin Cancer Res.

[46] Bray F, Ferlay J, Soerjomataram I, Siegel RL, Torre LA, Jemal A (2018). Global cancer statistics 2018: GLOBOCAN estimates of incidence and mortality worldwide for 36 cancers in 185 countries. CA Cancer J Clin.

[47] Locher C, Batumona B, Afchain P, Carrere N, Samalin E, Cellier C (2018). Small bowel adenocarcinoma: French intergroup clinical practice guidelines for diagnosis, treatments and follow-up (SNFGE, FFCD, GERCOR, UNICANCER, SFCD, SFED, SFRO). Dig Liver Dis.

[48] Montogomery EA, Yantiss RK, Snover DC, Tang LH (2017). Tumors of the intestines. AFIP atlas of tumor pathlogy, fourth series, fascicle 26.

